# Supplementary material for: Causal interplay between lactose intolerance and gut microbiota: a combined bidirectional Mendelian randomization and in vivo validation study
Source: Front Nutr. 2026 Jun 1;13:1803337. doi: 10.3389/fnut.2026.1803337 (PMC13265576; doi:10.3389/fnut.2026.1803337)
Supplement: Supplementary file 1 [file Data_Sheet_1.zip › supplementary materials/Forward/leave one out cross validation/ebi-a-GCST90027613.finngen_R12_E4_LACTONAS.pdf]

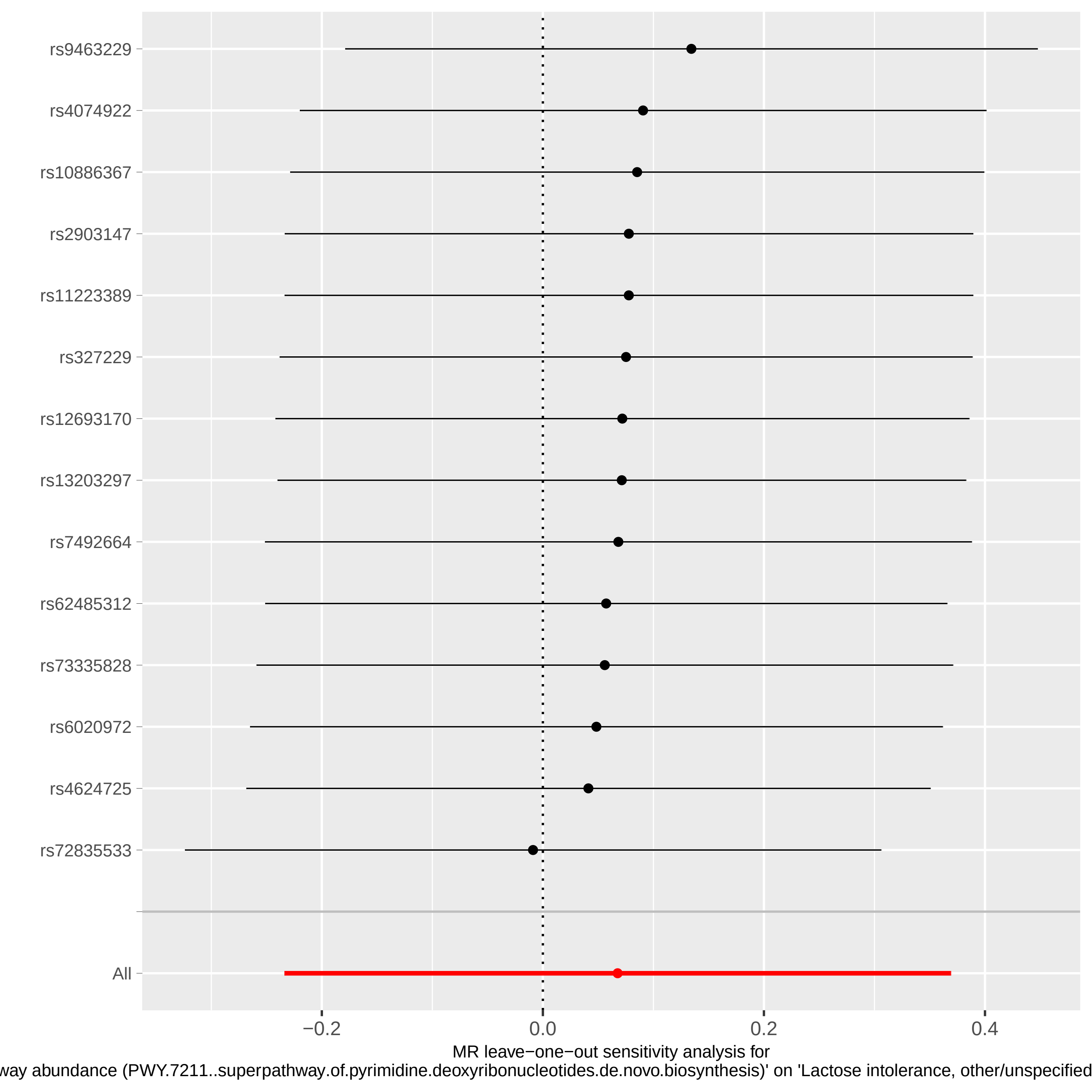
rs9463229
rs4074922
rs10886367
rs2903147
rs11223389
rs327229
rs12693170
rs13203297
rs7492664
rs62485312
rs73335828
rs6020972
rs4624725
rs72835533
All
−0.2
0.0
0.2
0.4
MR leave−one−out sensitivity analysis for
way abundance (PWY.7211..superpathway.of.pyrimidine.deoxyribonucleotides.de.novo.biosynthesis)' on 'Lactose intolerance, other/unspecified
